# Explaining cultural emotion in Chinese pop music with multimodal AI: educational and socio-emotional implications

**DOI:** 10.3389/fpsyg.2026.1734295

**Published:** 2026-04-09

**Authors:** Qilong Shi, Yan Zhou

**Affiliations:** 1 School of Music, Anshan Normal University, Anshan, Liaoning, China; 2 Department of Global Music, Graduate School, Kyonggi University, Suwon-si, Republic of Korea

**Keywords:** Chinese pop songs, emotional evolution, emotion recognition, multimodal machine learning, socio-cultural context

## Abstract

**Objective:**

This study investigates the evolutionary patterns of emotions in Chinese pop songs and examines how mainstream emotional characteristics align with shifting socio-cultural values, highlighting the implications for music education and culturally responsive pedagogy.

**Methods:**

We construct a corpus of Chinese pop songs from 1980 onward. Building on an improved Thayer emotion model, we introduce external affective resources to semantically enhance labels and apply semantic mapping to optimize manual annotations. We propose a multimodal emotion recognition model (M2ER) that integrates lyric and audio features to achieve fine-grained, segment-level annotation. Emotional evolution across periods is analyzed via distributional profiles, intensity dynamics, and association patterns.

**Results:**

The label optimization procedure improves the discriminability and representativeness of emotion categories. M2ER attains recognition accuracies of 84.49% (complete songs) and 78.65% (segments). We identify a historical trajectory from “lively and passionate” to “lyrical and gentle,” and subsequently to “diverse and delicate,” closely linked to broader socio-cultural transformations. Educational implications. By explaining which lyrical and acoustic cues underpin specific affective states and how these cues shift across eras, our findings offer actionable evidence for curriculum design (e.g., periodized listening modules), language-through-lyrics activities, and socio-emotional learning (SEL) in music classrooms. The corpus and interpretable analyses support culturally responsive teaching by situating repertoire choices within community histories and value change, and by scaffolding discussions of identity, emotion regulation, and aesthetic preference among diverse learners.

**Conclusion:**

Multimodal machine learning provides a new lens to understand public affect as expressed in popular music and to translate these insights into educational practice. The study deepens the cultural interpretation of emotion in music while informing school-based and community music education that aims to foster emotional well-being and inclusive engagement.

## 1 Introduction

Music is an important carrier of human emotional expression and is closely related to socio-cultural and public psychological states. As a form of popular culture, pop music is a direct reflection of the mainstream values, aesthetic tastes, and emotional appeals of a particular era (Liu and Wang, 2019). Chinese pop music has experienced a developmental journey from its emergence to prosperity since the 1980s. During this process, the styles and genres of pop music have continuously evolved, and the emotional connotations have become increasingly rich and diverse. In-depth analysis of the emotional evolution of Chinese pop music not only helps to interpret the social landscape of different historical periods from a musical perspective but also deepens our understanding of the developmental patterns of popular culture.

Emotional expression has always been a core issue in music research. Traditional music emotion research mainly adopts qualitative analysis methods, manually selecting representative works and exploring the emotions they convey from the perspectives of musicology and aesthetics (Juslin and Sloboda, 2001). However, this analysis approach based on subjective perceptions and experiences is often limited by the sample size, making it difficult to comprehensively depict the mainstream music emotions of an era. With the rise of digital humanities research paradigms, quantitative analysis and data mining techniques have gradually been introduced into music research, promoting the development of music emotion computing. Advances in music emotion recognition technology have made it possible to extract emotional features from massive music data, laying the foundation for macro-level music emotion analysis. However, current research in this area mainly focuses on Western classical music, and there is still a relative lack of systematic investigation of Chinese pop music. Moreover, existing studies mostly remain at the stage of static emotion classification, with insufficient attention paid to the dynamic evolution of music emotions.

In view of this, this study attempts to explore the evolutionary trajectory of emotions in Chinese pop music since the reform and opening up using the research paradigm of digital humanities. The main contributions are as follows: First, for the problem of emotion label mapping in pop music, an label semantic enhancement method is proposed. This method utilizes external emotion dictionaries and massive lyric corpora to significantly improve the discriminability of emotion labels and optimize the quality of manual annotations. Second, a multimodal emotion recognition model integrating lyric content and sound information is constructed, achieving fine-grained emotion annotation at the song segment level, providing an important data foundation for subsequent emotion evolution analysis. Third, the evolutionary patterns of Chinese pop music emotions are systematically analyzed at both macro and micro levels. At the macro level, the focus is on examining the overall distribution characteristics of music emotions and the diachronic trends of intensity changes for each emotion category. At the micro level, the aim is to reveal the patterns and evolutionary characteristics of emotional associations in different periods. On this basis, this study further explores the changes in the socio-cultural context reflected by the emotional changes in music.

The subsequent chapters of this paper are arranged as follows: Section 2 systematically reviews the current state of related research; Section 3 introduces the data sources and research methods used in this study; Section 4 reports the experimental results of multimodal emotion recognition; Section 5 analyzes the evolutionary patterns of Chinese pop music emotions from multiple perspectives; Section 6 summarizes the full text, discusses the limitations of the research, and outlines future research directions.

## 2 Related research

Music emotion recognition (MER) is one of the important research problems in the field of music information retrieval. Accurately capturing the emotions contained in music not only enriches the descriptive dimensions of music content but also provides support for applications such as personalized music recommendation and music therapy. The construction of emotion models is the theoretical basis for conducting MER research. The widely used music emotion models mainly include the Hevner model and the Thayer model. The Hevner model, from the perspective of music aesthetics, extracts 8 emotion categories with a total of 66 adjectives, covering a wide range but the logical relationships between categories are not yet clear (Zhang and Wei, 2025). The Thayer model, from the dimensional perspective of psychology, takes valence and arousal as coordinate axes and divides emotions into four quadrants: anxious and tense, angry and excited, sad and depressed, and calm and relaxed. This model has a concise structure and clearer differentiation between emotion categories, and has been widely applied in MER tasks (Kim et al., 2010). However, for Chinese music with rich emotions, directly applying existing models may be difficult to fully capture its emotional connotations, and it is necessary to make adaptive improvements to the model according to the characteristics of the research object.

Early MER research mainly constructed emotion recognition models based on acoustic features of audio signals (such as rhythm, pitch, timbre, etc.). Yang and Chen (2012) extracted low-level acoustic descriptors such as MFCC and spectral centroid, and used machine learning classifiers such as support vector machines (SVM) and Gaussian mixture models (GMM) to achieve good recognition results on Western classical music datasets. However, these methods usually require a large amount of feature engineering and are difficult to mine the intrinsic semantic features of music. With the development of deep learning technology, models such as convolutional neural networks (CNN) and long short-term memory networks (LSTM) have been introduced into MER tasks, showing advantages in automatically learning high-level feature representations. Malik et al. (2017) used a combination of CNN and LSTM based on Mel spectrograms, and the recognition accuracy exceeded traditional machine learning methods on two benchmark datasets.

Although acoustic features can reflect the emotional expression of music, the semantic information carried by lyrics cannot be ignored. In recent years, some studies have attempted to introduce lyric features into MER models to explore the possibility of multimodal emotion recognition. Malheiro et al. (2016) constructed a multimodal emotion annotation corpus containing lyrics and audio, used rule-based classifiers and SVM to model semantic features and acoustic features respectively, and fused the results of the two modalities at the decision level, achieving better recognition performance than single modalities. Delbouys et al. (2018) utilized deep learning techniques and designed an end-to-end multimodal MER model, using CNN to extract text features of lyrics and Mel spectrogram features of audio, and then using attention mechanisms to dynamically fuse the information of the two modalities, achieving fine-grained emotion annotation at the word and frame levels. However, existing research mostly regards MER as a static classification problem, with little attention paid to the dynamic evolution characteristics of music emotions. Wang et al. (2012) first proposed the concept of dynamic MER and used hidden Markov models (HMM) to capture the transition patterns of music emotions at different time scales. Xia et al. (2019) further considered the continuous change characteristics of music emotions, used sliding windows to extract acoustic features from audio, and used LSTM to model their temporal dependencies, realizing the prediction of continuous emotions. However, in general, fine-grained and dynamic MER analysis is still in its infancy, mainly limited by the scarcity of emotion annotation data with time labels.

In addition to the above studies, recent work has systematically explored machine learning techniques for music emotion classification and their sustainability implications. For example, Shelke and Patil proposed and evaluated several machine learning-based approaches to classify emotions from music signals and provided a comprehensive review of music emotion classification techniques, highlighting current challenges and opportunities for future development (Shelke and Patil, 2024). These studies further demonstrate the potential of data-driven models for music emotion recognition, but they mainly focus on Western datasets and static classification settings.

The above research mainly focuses on the content analysis methods of MER, while lacking a macro examination of the evolutionary patterns of music emotions. Current related explorations in this area are mainly concentrated in the field of ancient poetry. Ren (2016) used text mining techniques to analyze the distribution characteristics and diachronic evolution of emotions in more than 1,000 poems from the Tang and Song dynasties, combining the creative psychology of poets and the socio-cultural context to explain the reasons for the formation of the predominant emotional tones in different periods. These studies provide useful insights for the analysis of emotional evolution in pop music.

In view of this, this study intends to conduct explorations in the following aspects:

First, drawing on the ideas of poetry emotion research, examining the dynamic evolutionary patterns of Chinese pop music emotions at both macro and micro levels, while grasping the overall emotional distribution, excavating the diachronic trends of intensity changes for each emotion category and the patterns of emotional associations in different periods.
Second, for the specific object of Chinese pop music, making adaptive improvements to classic emotion models. In the process of model optimization, a label semantic enhancement method based on external knowledge bases is proposed, utilizing rich emotion vocabularies and lyric corpora to improve the quality of manual annotations and optimize the representational ability of the model.
Third, constructing a multimodal MER model that integrates lyric content and audio acoustic features to achieve fine-grained emotion annotation at the song segment level. To solve the problem of scarcity of fine-grained annotation corpora, exploring the possibility of transferring the model to learn on unlabeled data.
Fourth, from the perspective of digital humanities, correlating the evolution of music emotional characteristics in different eras with changes in the socio-cultural context, discussing the interactive relationship between music emotions and macro cultural trends and public emotions, and revealing the humanistic connotations behind the evolution of music emotions.

## 3 Data and methods

### 3.1 Data sources and preprocessing

This study takes Chinese pop music from 1980 onwards as the research object, with data sourced from the NetEase Cloud Music platform. NetEase Cloud Music is one of the largest online music platforms in China, with a very large registered user base and extensive coverage of mainstream Chinese pop repertoire across different eras and genres. Therefore, although our data come from a single platform, they can still capture a broad and representative sample of the contemporary Chinese pop music landscape.

Through a Python web crawler program, we obtained the metadata and audio files of 8,562 pop songs from 1980 to 2024. The metadata includes information such as song name, singer, album, release time, and lyrics.

In the data preprocessing stage, we first performed deduplication and cleaning of the raw data, removing samples with incomplete metadata or missing audio. Then, we used the jieba word segmentation tool to segment the lyric text and removed stop words and punctuation marks in the lyrics according to a customized stop word list. For the audio data, we used the librosa library to extract the first 30 s of each song^1^, with a sampling rate of 22,050 Hz, and converted it to mono, 16-bit WAV format. This yields 7,628 valid song samples after cleaning, derived from 8,562 initially crawled pop songs from 1980 to 2024 from NetEase Cloud Music.

To examine the phased changes in the emotional characteristics of pop music, referring to the era division method of (Chen et al., 2006), we divided the dataset into four time periods: 1980–1990 (917 songs), 1991–2000 (2,115 songs), 2001–2010 (3,220 songs), and 2011–2024 (1,376 songs). These four time periods correspond to the starting, developing, prospering, and diversifying stages of Chinese pop music, representing different sociocultural backgrounds and aesthetic tastes.

### 3.2 Emotion label optimization

The Thayer emotion model is a widely adopted emotion classification framework in MER research, but due to the differences in emotional expression between Chinese and Western music, the applicability of this model in Chinese music research needs further verification.

To better capture the rich emotional connotations of Chinese pop music, based on the four quadrants (Q1-Q4) of the original Thayer model, according to the suggestions of musicians and psychologists, this study added two emotion categories^2^: confiding and lyrical (Q5) and passionate and high-spirited (Q6). Among them, Q5 corresponds to songs that express emotions such as sentimentality, nostalgia, and melancholy, while Q6 corresponds to songs that express uplifting, enterprising spirits. These two types of emotions occupy an important proportion in Chinese pop music but are not reflected in the original Thayer model. While expanding the emotion categories, we also referred to the Hownet emotion dictionary and merged semantically similar words in the original model, forming an improved Thayer model containing 42 emotion words (Table 1).

**Table 1:** Improved Thayer emotion model.

Number	Emotion category	Emotion words
Q1	Anxious and tense	Tense, irritable, uneasy, fearful, anxious, confused, hesitant, perplexed
Q2	Angry and excited	Angry, indignant, furious, excited, fanatical, thrilled
Q3	Sad and depressed	Sad, sorrowful, distressed, heartbroken, painful, depressed, desperate, helpless
Q4	Calm and relaxed	calm, serene, soothing, warm, tranquil, peaceful, cheerful, happy
Q5	Confiding and lyrical	Confiding, lyrical, sentimental, melancholic, nostalgic, emotional, tender, lingering
Q6	Passionate and high-spirited	Passionate, high-spirited, uplifting, enterprising, heroic, inspiring, confident, determined

In the manual music annotation process, we found that the original labels had problems such as being overly concentrated or divergent, having high ambiguity, and being strongly subjective, making it difficult to ensure the consistency of annotation quality. Therefore, this study proposes a label optimization method based on external knowledge. First, in a large-scale corpus containing 10 million lyrics, the co-occurrence words of each original label are counted, that is, other emotion words that frequently appear in the context window (window size of 5) of the label. Co-occurrence words contain the contextual semantic information of the original labels and can be used to refine and expand the emotional connotations of the labels. Next, the Hownet emotion dictionary is used to calculate the semantic similarity between the original labels and the emotion categories in the improved Thayer model. Hownet contains more than 50,000 emotion words and their semantic relationships, which can accurately depict the emotional tendencies between words. We use cosine similarity to measure the semantic distance between the original labels and each category, and combine the label co-occurrence word information to determine the mapping category of the original labels in the Thayer model. If the similarity between the original label and all categories is lower than the threshold (0.5), it is classified as the “other” category (Q0).

For example, we found that “sentimental” often co-occurs with words such as “melancholy,” “nostalgic,” and “emotional,” and belongs to the synonyms of words such as “sad” and “sorrowful” in Hownet, so “sentimental” can be mapped to the Q3 category (sad and depressed) in the Thayer model. Through this mapping optimization, more than 90% of the original labels were given clear affiliations. The optimized emotion labels are more in line with the classification logic of the Thayer model and retain the emotional characteristics of Chinese pop music, avoiding the problems of labels being overly concentrated or divergent, and enhancing the discriminability and representativeness of the labels.

### 3.3 Multimodal emotion recognition

**Figure 1:**
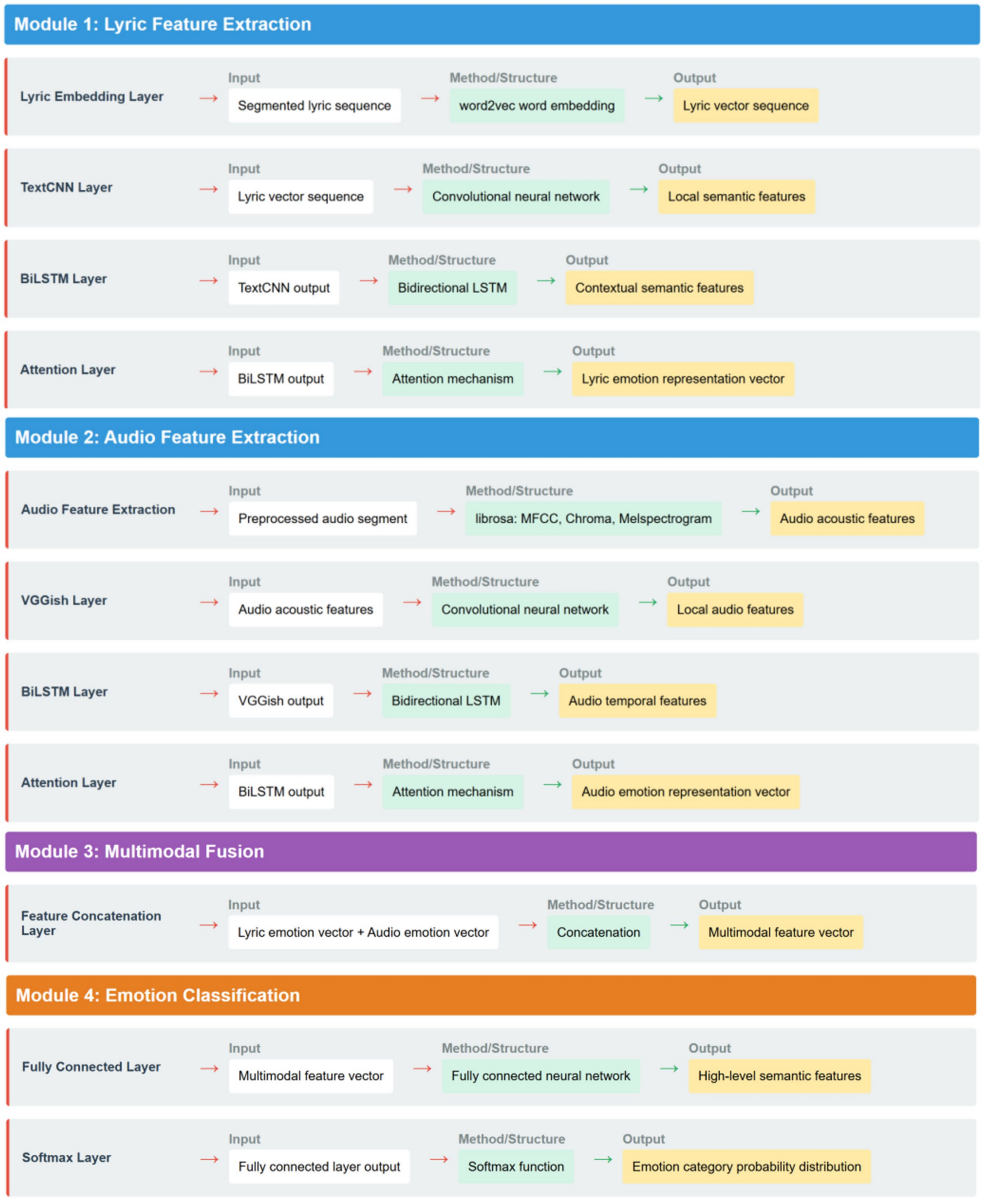
Structural composition of the M2ER model.

The emotional expression of Chinese pop songs relies on the joint effect of lyric content and melodic sound. Lyrics carry the thematic ideas and emotional connotations of the songs, while elements such as rhythm, timbre, and intensity in the audio create a specific emotional atmosphere. Considering the synergistic effect of lyrics and audio, this study constructs an emotion recognition model M2ER that integrates the two modalities (Figure 1). This model includes three core modules: lyric feature extraction, audio feature extraction, and multimodal emotion classification.

The purpose of the lyric feature extraction module is to learn the semantic representation of the lyric text. We first use the word2vec model to convert each lyric word into a 200-dimensional word vector, thereby representing the lyric text as a vector sequence. Next, a TextCNN structure consisting of three convolutional layers (with kernel sizes of 2, 3, and 4) and a max-pooling layer is used to extract local features of lyric segments. Then, the output of TextCNN is fed into a bidirectional LSTM network (BiLSTM) to learn the contextual information and long-distance semantic dependencies of the lyrics. BiLSTM encodes the lyric vector sequence in the forward and backward directions, enabling better capture of the global semantics of the lyrics. Finally, we connect an attention mechanism layer after BiLSTM to automatically calculate the relevance weights between each position in the lyric sequence and the emotion labels, highlighting the key segments in the lyrics that are closely related to emotional expression, and obtaining an emotion-oriented representation vector of the lyrics.

The audio feature extraction module aims to mine the emotional information contained in the audio signal. We used the librosa library to extract acoustic features such as Mel frequency cepstral coefficients (MFCC), chroma frequency (Chroma), and Mel spectrogram (Melspectrogram) for each 30-s audio segment. Among them, MFCC describes the micro characteristics of audio such as timbre and pitch, Chroma reflects the macro characteristics of audio such as tone and harmony, and Melspectrogram shows the energy distribution of the audio in the time-frequency dimension. We input the extracted features into a pre-trained VGGish network (a variant of the VGG image classification model) to learn local audio features through convolution and pooling operations. Next, similar to lyric feature extraction, we use BiLSTM to perform sequence modeling on the output of VGGish, and focus on emotion-related audio segments through an attention mechanism to obtain an audio feature vector containing emotional information.

The multimodal emotion classification module fuses the lyric features and audio features to predict the emotion category of the song. We first concatenate the features of the two modalities in the hidden layer to obtain a joint representation vector. Then, the vector is input into a fully connected network (FCN) to extract high-level semantics through non-linear transformations. The output of the FCN is transformed into a probability distribution of emotion categories through a softmax function. We use the cross-entropy loss function to measure the difference between the predicted distribution and the true distribution, and use the Adam optimizer to minimize the loss function and iteratively update the model parameters. The M2ER model takes complete songs as input during the training stage and outputs the emotion category they belong to. In the application stage, it takes song segments (30 s) as input and predicts the emotion distribution of each segment, achieving more fine-grained emotion recognition.

It should be noted that there is currently a lack of large-scale emotion annotation data for song segments. To solve this problem, this study adopts a transfer learning method to apply the M2ER model trained on complete songs to unlabeled song segments. We assume that complete songs and song segments have a consistent distribution in the feature space, and the feature-emotion mapping relationship learned by the model can be transferred across granularities. Therefore, using M2ER to directly predict the emotion distribution of song segments can achieve unsupervised segment annotation. While alleviating annotation costs, this method also provides data support for dynamic emotion analysis.

### 3.4 Emotion evolution analysis

This study analyzes the evolution patterns of emotions in Chinese pop music from three levels: (1) changes in overall emotion distribution; (2) trends of changes in the intensity of individual emotions; (3) emotional association patterns of different periods.

First, we counted the number and proportion of songs in each emotion category in each time period to depict the overall characteristics of the emotion distribution of pop music in different periods. By calculating the information entropy of emotion categories, the changes in emotional diversity in each period can be measured. To reveal the statistical significance of changes in emotion distribution, we used the chi-square test method, with the time period as the independent variable and the emotion category as the dependent variable, to test whether the differences in emotion distribution in different periods are significant.

Second, we examined the diachronic trends of intensity changes for each emotion category. Taking the emotion probability distribution output by the M2ER model as the association strength between songs and various emotions (ranging from [0, 1], with larger values indicating stronger association), we calculated the mean and standard deviation of the intensity of each emotion category in each period, depicting the development trends of different emotions at the macro level. To explore the significance of changes in emotion intensity, for each pair of adjacent periods of the same emotion category, we used the Mann–Whitney U test to compare the differences in their intensity distributions and examine the phased characteristics of emotion intensity.

Finally, we mined the emotional association patterns of different periods. Taking emotion categories with occurrence probabilities greater than 0.5 as the emotional labels of each song, we counted the frequently co-occurring emotion combinations in each period, and used the Apriori association rule learning algorithm, with a minimum support of 0.01 and a minimum confidence of 0.5 as thresholds, to discover the association rules between different emotions. An association rule is of the form “A->B (support=0.2, confidence=0.8),” indicating that among all songs with emotion A, 20% also have emotion B, and among all songs with emotion B, 80% have emotion A. Through association rules, we can infer the dominant emotional logic of different periods and explain the internal reasons for the occurrence of specific combinations.

In summary, this study used web crawlers to obtain large-scale Chinese pop music data, which were divided into four stages according to time span. Based on the Thayer emotion model, the original emotion labels were optimized by combining external knowledge bases, improving the quality of manual annotations. Subsequently, we designed a multimodal emotion recognition model that integrates lyric and audio features, and achieved fine-grained annotation of song segments through transfer learning. Finally, from the perspectives of overall distribution, independent intensity, and association patterns, this study used statistical tests, association rules, and other methods to quantitatively analyze the evolution patterns of pop music emotions. The research process is shown in Table 2.

**Table 2:** Research process.

Stage	Task	Method	Input	Output
Data acquisition	Crawling song metadata and audio	Web crawler	NetEase Cloud Music song pages	Song metadata (title, singer, album, release time, lyrics, etc.) and audio files
Data preprocessing	Metadata cleaning	Data deduplication, missing value processing	Raw song metadata	Deduplicated, complete song metadata
	Lyric segmentation	Jieba segmentation, stop word removal	Raw lyric text	Segmented lyric sequence
	Audio conversion	Extracting first 30 s, converting to mono, 16-bit, 22,050 Hz sampling rate WAV format	Raw audio files	Preprocessed audio segments
Label optimization	Label semantic enhancement	Calculating semantic similarity between original labels and emotion words in dictionary	Original labels, external emotion dictionary	Emotional tendency descriptions of labels
	Label mapping	Combining semantic similarity and co-occurrence information to map original labels to improved Thayer model	Original labels, semantic similarity, co-occurrence information	Optimized emotion labels
Feature extraction	Lyric feature extraction	Word vector representation, TextCNN extracting local features, BiLSTM learning contextual information, attention mechanism highlighting key segments	Segmented lyric sequence	Lyric emotional semantic representation vector
	Audio feature extraction	Extracting MFCC, Chroma, Melspectrogram, etc. acoustic features, VGGish learning local audio features, BiLSTM modeling temporal information, attention mechanism focusing on emotion-related segments	Preprocessed audio segments	Audio feature vector containing emotional information
Emotion recognition	Multimodal feature fusion	Concatenating lyric features and audio features	Lyric feature vector, audio feature vector	Multimodal fused features
	Emotion classification	Fully connected network + Softmax layer, predicting probability distribution of multiple emotion categories	Multimodal fused features	Probability distribution of emotion categories (complete songs) / Emotion distribution of song segments (song segments)
Emotion evolution analysis	Overall distribution analysis	Counting proportion of emotion categories in each period, chi-square test analyzing significance of differences	Emotion labels of songs in each period	Overall characteristics and evolution patterns of emotion distribution
	Independent intensity analysis	Calculating mean emotion intensity of each period, Mann–Whitney U test analyzing significance of intensity differences between adjacent periods	Emotion intensity distribution of songs in each period	Diachronic trends of intensity changes for different emotions
	Association pattern analysis	Frequent pattern mining, Apriori association rule learning algorithm	Emotion label combinations of songs in each period	Frequent emotion combination patterns and association rules in different periods

## 4 Emotion recognition experiment

### 4.1 Evaluation metrics

To comprehensively evaluate the performance of the model in the pop music emotion recognition task, this study uses four common metrics: accuracy, precision, recall, and F1-score. These metrics are defined and calculated based on the confusion matrix.

The confusion matrix is a matrix that summarizes the performance of a classification model. By comparing the model prediction results with the true labels, four key quantitative indicators can be obtained:

True Positive (TP): the number of positive class samples that are correctly predicted as positive.
False Positive (FP): the number of negative class samples that are incorrectly predicted as positive, i.e., actually negative but predicted as positive.
False Negative (FN): the number of positive class samples that are incorrectly predicted as negative, i.e., actually positive but predicted as negative.
True Negative (TN): the number of negative class samples that are correctly predicted as negative.

Based on these four indicators, the following four metrics for evaluating model performance can be calculated:

(1) Accuracy: the proportion of the number of samples correctly predicted by the model to the total number of samples, calculated as:
Accuracy = (TP + TN) / (TP + FP + TN + FN).
Accuracy intuitively reflects the overall performance of the model, but in the case of class imbalance, accuracy may not well reflect the actual effect of the model.

(2) Precision: the proportion of samples predicted as positive by the model that are actually positive, calculated as:
Precision = TP / (TP + FP).
Precision reflects how confident the model is in determining negative samples as positive. The higher the precision, the more cautious the model’s predictions are.

(3) Recall: the proportion of actual positive samples that are correctly predicted as positive by the model, calculated as:
Recall = TP / (TP + FN).
Recall reflects the ability of the model to recognize positive samples. The higher the recall, the stronger the model’s ability to recognize positive samples.

(4) F1-score: the harmonic mean of precision and recall, calculated as:
F1 = 2 × Precision × Recall / (Precision + Recall).

It can be seen that the F1-score considers both the precision and recall of the classification and is a comprehensive evaluation metric. The higher the F1-score, the better the balance the model achieves between precision and recall.

For multi-classification tasks, the calculation methods of the above four metrics are slightly different. There are two common processing methods:

Macro-averaging: first calculate the metrics for each category separately, and then take the arithmetic mean of the metrics of each category as the overall metric. This method treats each category equally, but may be affected by the number of samples in small categories.
Micro-averaging: accumulate the TP, FP, TN, and FN values of all categories, and then calculate the metrics based on the accumulated values. This method comprehensively considers all samples and focuses more on overall performance, but may be affected by the number of samples in large categories.

This study uses the macro-averaging calculation method to evaluate the performance of the model on each emotion category in a balanced manner. By comparing the accuracy, precision, recall, and F1-score of different models, their advantages and disadvantages in the pop music emotion recognition task can be evaluated from multiple perspectives.

### 4.2 Experimental results

This study divides the dataset into training and test sets in a ratio of 8:2, and conducts comparative experiments on the proposed multimodal model (M2ER) and three baseline models:

BOW+SVM: representing lyrics with a bag-of-words (BOW) and classifying with a support vector machine (SVM).
TextCNN+BiLSTM: a variant of M2ER that only uses lyric features.
VGGish+BiLSTM: a variant of M2ER that only uses audio features.

Table 3 shows the performance of each model on the test set. It can be seen that:

**Table 3:** Model performance comparison.

Model	Accuracy	Precision	Recall	F1
BOW + SVM	70.26%	69.52%	71.34%	70.42%
TextCNN + BiLSTM	79.73%	80.01%	78.67%	79.33%
VGGish + BiLSTM	76.64%	75.29%	77.86%	76.55%
M2ER	84.49%	85.12%	83.62%	84.36%

The M2ER model that fuses lyric and audio information significantly outperforms models that only use a single modality, confirming the effectiveness of multimodal learning.

TextCNN+BiLSTM and VGGish+BiLSTM based on deep learning outperform the shallow bag-of-words representation and traditional machine learning classifiers, respectively, indicating that deep neural networks can better mine the intrinsic emotional features of music.

In the multimodal emotion recognition experiment, the dataset of 7,628 songs was randomly divided into a training set and a test set with a ratio of 8:2 at the song level, ensuring that no song appeared in both sets.

And all baselines, such as BOW+SVM, TextCNN+BiLSTM, and VGGish+BiLSTM, were trained under the same train–test split and comparable optimization settings. So that the reported performance differences, with accuracy and F1-score reaching 84.49 and 84.36%, respectively, verifying the effectiveness of the model in the pop music emotion recognition task.

During the application stage, each song is segmented into 30-s units and fed into the trained M2ER model to obtain the emotion probability of each segment. To evaluate transferability from song-level to segment-level, we randomly sampled 300 segments for manual annotation and compared them with model predictions. The results show that the accuracy of the M2ER model in the segment emotion recognition task is 78.65%, and the F1-score is 77.22%, confirming that the model trained on complete song annotation data can be well transferred to the emotion recognition task of song segments, providing a reliable data foundation for subsequent emotion evolution analysis.

In general, by setting reasonable evaluation metrics and conducting detailed experiments on the Chinese pop music dataset, this section systematically evaluated the performance of the multimodal emotion recognition model M2ER, confirming the effectiveness of modeling by integrating lyric content and audio acoustic features. At the same time, this section also explored the feasibility of training models using complete song annotation data and transferring them to the emotion recognition task of song segments, laying a good foundation for subsequent emotion evolution analysis. The excellent performance of the M2ER model on key metrics such as accuracy and F1-score fully reflects the application value of multimodal machine learning techniques in the field of pop music emotion computing.

## 5 Emotion evolution analysis

### 5.1 Overall emotion distribution

Table 4 shows the emotion distribution of Chinese pop music in four periods, clearly presenting the percentages of the six emotion categories in each period.

**Table 4:** Emotion distribution of pop music in different periods.

Emotion category	1980–1990	1991–2000	2001–2010	2011–2024
Q1 (anxious and tense)	7.3%	8.6%	12.9%	15.2%
Q2 (angry and excited)	5.1%	6.7%	8.3%	13.7%
Q3 (sad and depressed)	6.9%	12.5%	20.6%	29.4%
Q4 (calm and relaxed)	28.4%	24.2%	22.8%	19.5%
Q5 (confiding and lyrical)	16.7%	28.3%	26.4%	18.1%
Q6 (passionate and high-spirited)	35.6%	19.7%	9.0%	4.1%

As shown in Table 4, the distribution of emotion categories is imbalanced, with some emotions (e.g., Q3 “sad and depressed” and Q5 “confiding and lyrical”) occurring more frequently than others (e.g., Q2 “angry and excited”). To mitigate the impact of this imbalance on model training, we adopted a class-weighted cross-entropy loss, where the weight of each emotion category was set inversely proportional to its frequency in the training set. In addition, we report macro-averaged precision, recall, and F1-score, which treat each category equally and thus provide a more balanced evaluation under imbalanced labels.

And from the data, some main trends of change can be seen:

The proportion of passionate and high-spirited (Q6) emotions decreased significantly from 35.6% in the 1980s to 4.1% in the past decade, reflecting a significant change in people’s emotional expression during this period.
The proportion of sad and depressed (Q3) emotions continued to rise from 6.9% in the 1980s, reaching a peak of 29.4% in 2011–2024, becoming the main emotional tone of this period.
The calm and relaxed (Q4) emotions show an overall downward trend, but the change is relatively gradual, indicating that this emotion has always been an indispensable component of pop music.
The confiding and lyrical (Q5) emotions reached a peak of 28.3% in the 1990s, after which they declined but still maintained a relatively high level of around 18%.
The anxious and tense (Q1) and angry and excited (Q2) emotions show an overall upward trend in their proportions, especially a significant increase after entering the new century, reflecting the influence of increasing social pressure on people’s emotions.

Combining the socio-cultural background of each period, a more in-depth interpretation of these emotional distribution changes can be made:

The 1980s marked the early stage of reform and opening up, and song emotions were mainly positive and uplifting. On the one hand, people were full of anticipation for new life, and a large number of works praising reform and new era emerged, such as “Story of Spring,” reflecting the spirit of the times of vigorous progress. On the other hand, the Chinese music market was not yet fully open, and the number of popular songs was limited, mostly dominated by lyrical folk songs, such as “In That Distant Place,” showing simple and peaceful emotions.

In the 1990s, pop music began to develop diversely, and emotional expressions became richer, but overall, they were still dominated by positive and upbeat emotions. The proportion of confiding and lyrical songs increased the most significantly, with representative works such as “Friends” and “The Sea,” expressing people’s yearning and reflection on ideals, friendship, and love. At the same time, some songs also began to express emotions of confusion and loss, but they had not yet become mainstream.

Entering the 21st century, with the rapid economic development and social transformation, people’s emotional needs have become increasingly diverse. Songs with sad and melancholic themes have increased significantly, such as “Mr. Lonely” and “Those Adventures You Dreamed Of,” reflecting the pressures and perplexities faced by modern people. At the same time, some songs have also expressed dissatisfaction and criticism of real-life issues, showing signs of a rebellious spirit.

In the past decade, pop music has gradually moved away from monotonous and one-sided tendencies, and emotional expressions have become more three-dimensional and rich. On the one hand, the process of urbanization has intensified the pressures and anxieties of modern people, and sad songs have continued to grow. On the other hand, the liberation of individuality and the awakening of self-awareness have led more singers to express personal emotions, and the proportion of confiding and lyrical songs has increased. At the same time, some songs directly confront social issues and express emotions of anger and dissatisfaction.

Overall, the mainstream emotions of Chinese pop music have undergone a transformation from “revolutionary optimism” to “melancholy and sadness” and then to “delicate and diverse,” vividly reflecting the profound influence of social changes on public emotions in different periods. This trajectory of change is highly consistent with the process of China’s 40 years of reform and opening up and social transformation, reflecting the social mapping function of pop music as a carrier of popular culture. Through the examination of macro-emotional distribution, one can glimpse the social landscape of different eras and gain insight into the evolution of the public emotional structure, highlighting the unique value of emotional computing methods in music sociology research.

### 5.2 Independent emotion intensity

Table 5 shows the average intensity values of the six emotion categories in four periods. The range of emotion intensity values is [0,1], and the larger the value, the stronger the emotion is expressed in the pop songs of the corresponding period.

**Table 5:** Mean intensity of various emotions in different periods.

Emotion category	1980–1990	1991–2000	2001–2010	2011–2024
Q1 (anxious and tense)	0.18	0.15	0.35	0.57
Q2 (angry and excited)	0.13	0.11	0.23	0.41
Q3 (sad and depressed)	0.21	0.19	0.52	0.66
Q4 (calm and relaxed)	0.45	0.43	0.41	0.39
Q5 (confiding and lyrical)	0.36	0.47	0.54	0.62
Q6 (passionate and high-spirited)	0.68	0.45	0.28	0.15

From this table, we can more intuitively see the diachronic trends of changes in the intensity of various emotions:

The intensity of anxiety (Q1) emotions was at a low level in 1980–2000, but increased significantly after entering the 21st century, reaching the highest value of 0.57 in 2011–2024.

The intensity of anger (Q2) emotions shows an overall upward trend, especially a more significant increase after 2001, rising from 0.13 in 1980–1990 to 0.41 in 2011–2024.

The intensity of sadness (Q3) emotions changed significantly, remaining at a low level before 2000, increasing sharply after 2001, and reaching the highest value of 0.66 in 2011–2024.

The intensity of calmness (Q4) emotions remains relatively stable, fluctuating within the range of 0.39–0.45.

The intensity of confiding (Q5) emotions shows a gradual upward trend, rising from 0.36 in 1980–1990 to 0.62 in 2011–2024.

The intensity of passion (Q6) emotions shows a significant downward trend, decreasing from the highest value of 0.68 in 1980–1990 to the lowest value of 0.15 in 2011–2024.

The intensity of passion (Q6) emotions reached its peak in the 1980s and then gradually declined and stabilized. This reflects people’s high enthusiasm for reform and opening up at the end of the 20th century, as well as the trend of praise and eulogizing themes gradually fading with the advancement of commercialization and secularization.

The intensity of anxiety (Q1) and sadness (Q3) emotions slightly decreased in the 1990s, but continued to rise after entering the 21st century, reaching a peak in the past decade. This change is closely related to the increased individual pressures during the social transformation period. Modern people face multiple dilemmas such as employment, housing, and interpersonal relationships, and negative emotions have accumulated, urgently needing an outlet for catharsis.

The intensity of confiding (Q5) emotions shows a continuous upward trend, reflecting that personal emotional expressions are receiving increasing attention in pop music. With the awakening of self-awareness, people are no longer satisfied with grand narratives, but pay more attention to individual life experiences and emotional experiences. Lyrical songs, with their characteristics of being close to life and resonating with listeners, have been favored by the public.

The intensity of calmness (Q4) emotions remains relatively stable overall in the four periods, indicating that soothing and comforting have always been indispensable functions of pop music. No matter how times change, people always need to obtain spiritual solace from music.

The intensity of anger (Q2) emotions is at a low level in all periods. This may be because mainstream music creation still intentionally avoids negative emotions. Although some songs express dissatisfaction with reality, truly anger-filled works are still relatively rare. At the same time, driven by commercialization, pop music tends to cater to popular tastes and avoid overly extreme emotional expressions.

The above analysis shows that with the development of the times, the collective emotions of grand narratives in Chinese pop music have weakened, and contradictory and complex individual emotions have increased, with personalized and diverse emotional expressions becoming mainstream. This change reflects, to a certain extent, the more diverse value concepts in Chinese society and the important influence of the awakening of personal self-awareness on public emotions. Based on the dynamic examination of emotional intensity, a new perspective is provided for understanding the internal causes of changes in music emotions. Combined with the analysis of macro-emotional distribution, it can not only reveal the overall trend of emotional evolution but also grasp the differential characteristics of emotional intensity at different stages, reflecting the unique advantages of quantitative analysis methods.

### 5.3 Emotional association patterns

Table 6 lists the five emotional association rules with the highest confidence in each period, revealing the dynamic combination characteristics of pop music emotions.

**Table 6:** Emotional association patterns in different periods.

Period	Association rule	Confidence
1980s and 1990s	passionate → calm	87%
	calm → confiding	83%
	confiding → sad	76%
	calm → passionate	72%
	passionate → confiding	65%
2001–2010	calm → confiding	79%
	confiding → calm	74%
	confiding → sad	69%
	anxious → sad	65%
	sad → confiding	62%
2011–2024	calm → confiding	82%
	confiding → anxious	73%
	sad → anxious	66%
	passionate → angry	55%
	confiding → angry	51%

In the 1980s and 1990s, positive and optimistic emotional combinations dominated, such as “passionate → calm,” “calm → confiding,” etc., but there were also some lyrical and sentimental combinations, such as “confiding → sad.” Overall, this period was dominated by positive and upbeat emotional combinations, interspersed with occasional melancholic emotions, reflecting the mainstream value pursuits of society in the early stage of reform and opening up, as well as the intertwining of people’s yearning for a better life and perplexed emotions in the midst of drastic changes.

Entering the 21st century, negative emotional combinations have increased significantly, such as “anxious → sad,” “sad → confiding,” etc. This indicates that in the pain of social transformation, people generally feel uncertain about the future and exhausted, and the emotions of anxiety, confusion, and melancholy are increasing day by day. At the same time, some songs try to point out a way for people, such as the combination of “confiding → calm,” reflecting the contradictory and complex social mentality and people’s yearning for emotional comfort.

In the past decade, personalized emotional expressions have become mainstream, such as the emergence of combinations like “confiding → anxious,” “confiding → angry,” which not only show the anxiety and unease of individuals but also reflect dissatisfaction and criticism of real-life issues. Sad and melancholic emotions have further intensified, becoming the prominent feature of this period. At the same time, passionate and angry emotional combinations have also risen, such as “passionate → angry,” reflecting the rebellious consciousness and critical spirit of contemporary youth.

It can be seen that with the development of the times, people’s emotional needs in music have become increasingly diverse, with complex emotions of positivity and negativity, individuality and society intertwined and coexisting, becoming the main feature of pop music in the new era. On the one hand, the expression of personal emotions has received more attention, and people no longer avoid inner emotions of sadness, confusion, and anxiety, but directly face reality and vent their dissatisfaction. On the other hand, criticism of social issues and the pursuit of ideals are still the main themes of some songs. The evolution of emotional association patterns in pop music vividly reflects the trajectory of changes in Chinese social thoughts, highlighting the profound influence of the rise of personal consciousness on public emotions. With the help of data mining methods to reveal the high-frequency emotional combinations in different periods and explore the social and cultural implications behind them, the dynamic association characteristics of pop music emotions are intuitively presented, providing new methodological support for traditional music sociology research.

Combining the above analysis of macro distribution, independent intensity, and association patterns at three levels, it can be seen that: Based on the research perspective and quantitative analysis methods of digital humanities, this study examined the patterns and characteristics of the evolution of Chinese pop music emotions from multiple angles and in an all-round way, revealing not only the overall trajectory of changes in pop music emotions but also depicting in detail the dynamic characteristics of emotional distribution, intensity, and association in different periods. By incorporating the changes in music emotions into a broader socio-cultural context, this study further clarifies the profound influence of mainstream value concepts, ideologies, and aesthetic tastes in different periods on public music emotions, as well as the mapping and leading role of pop music emotions on social culture, deepening the understanding of the interactive relationship between music emotions and society. It highlights the important value of combining emotion computing and digital humanities for music sociology research, and provides an inspiring example of the deep integration of traditional humanities and cutting-edge computational methods.

## 6 Conclusions and discussion

Taking Chinese pop music since 1980 as the research object, this study constructs a multimodal emotion recognition model from the two dimensions of lyric content and audio acoustics. Based on the recognition results of the model, this study systematically analyzes the distribution characteristics, intensity changes, and association patterns of pop music emotions in different periods, and relates the trajectory of emotional evolution to the process of Chinese social changes, exploring the interactive relationship between pop music emotions and socio-cultural contexts.

The research finds that the emotional distribution of Chinese pop music has undergone an evolutionary trajectory from “revolutionary optimism” to “melancholy and sadness” and then to “diverse and delicate.” This process of change is a reflection of the social transformation of China since the reform and opening up, vividly reflecting the changes in dominant values and collective emotions at different historical stages. In the longitudinal examination of emotional intensity, we find that positive emotions such as passion and optimism show a gradually fading trend, while the intensity of negative emotions such as sadness and anxiety continues to rise, and personalized emotional expressions are increasingly becoming mainstream. This change in the rise and fall of emotional intensity, to a certain extent, reflects the pain of the social transformation period, as well as the influence of factors such as increased individual pressure and the awakening of self-awareness on public emotions. From the dynamic changes in emotional association patterns, positive emotional combinations have gradually decreased since the 21st century, while contradictory and critical compound emotional combinations have become more common, reflecting the more diverse value concepts and the coexistence of contradictory emotions in the psychological state of society during the era of change. Overall, the evolutionary trajectory of Chinese pop music emotions is closely related to the pulse of Chinese social development, forming a bidirectional interaction and mutual reflection relationship: On the one hand, changes in music emotions profoundly reflect the ups and downs of public emotions in different eras, and on the other hand, pop music leads the changes in social and cultural trends through emotional expressions, playing an important role in shaping public emotions.

This study attempts to use the research paradigm and computational analysis methods of digital humanities to quantitatively depict the evolutionary patterns of pop music emotions from multiple angles, and discusses the social and cultural implications behind them, expanding the ideas and methods of music sociology research to a certain extent. However, limited by research perspectives and data characteristics, this study still has some limitations. First, although this study examines the influence of lyric content and audio acoustic features on emotional expression, it does not comprehensively include factors such as music style and song structure. In fact, the alternation of music genres itself may also have an important impact on emotional expression. In the future, we can try to introduce multi-dimensional features such as music style classification and singer identity recognition to enrich the examination perspective of emotional influencing factors. Moreover, all songs in this study were collected from a single online platform, NetEase Cloud Music, which may lead to potential sampling bias with respect to offline music consumption, niche subcultures, or other platforms with different user demographics. Future work will expand the dataset to multiple streaming services and distribution channels to further improve representativeness. Thirdly, this study is limited to the context of Chinese pop music and lacks a comparative perspective with Western or other Asian pop music scenes. We did not conduct genre-stratified analyses (e.g., comparing ballads, rock, or rap separately), nor did we perform cross-cultural comparisons. Future research can construct parallel datasets across genres and cultures, and examine whether the evolution patterns observed here are specific to Chinese pop music or reflect broader global trends in popular culture. In addition, although this study constructs a multimodal emotion recognition model based on deep learning and achieves good performance on the Chinese pop music dataset, there is still room for further optimization. In the future, we can try to introduce cutting-edge artificial intelligence technologies such as text representation learning and transfer learning to further improve the generalization performance and robustness of the model and enhance the reliability of the research conclusions. Finally, the generation and dissemination of pop music emotions are inseparable from the active participation of the audience. The audience’s perception, empathy, and feedback on music emotions have a direct impact on the popularity and prevalence of songs. In the future, we can consider mining the associations between music emotion labels and audience interaction behaviors (such as comments, reposts, favorites, etc.). On the one hand, it verifies the consistency between emotion recognition results and public emotion perception, and on the other hand, it explores the influence of music emotions on audience behavior. This not only expands the application value of this research (such as emotion labels guiding music recommendation, audience profile analysis assisting precise marketing, etc.) but also provides a new perspective for understanding the mechanism of music emotion dissemination.

Beyond music sociology, the proposed multimodal framework and large-scale temporal analysis also have potential implications for music therapy and affective computing. By capturing the fine-grained evolution of emotions within and across songs, the model can help identify segments that are suitable for different therapeutic purposes such as emotion regulation, relaxation, or emotional catharsis, especially in Chinese-speaking populations. At the same time, the M2ER framework and emotion evolution patterns can inform the design of affective computing systems, including emotion-aware music recommendation, intelligent tutoring systems that adapt musical content to learners’ emotional states, and interactive applications that respond to users’ socio-emotional needs. Although the present study is not based on clinical samples, it provides a data-driven foundation for future interdisciplinary work that integrates music therapy, education, and affective computing.

In general, this study utilizes the perspective and methods of digital humanities to quantitatively analyze the dynamic evolution patterns of Chinese pop music emotional expressions. While revealing the interactive relationship between music emotions and social culture, it makes a beneficial attempt to enrich the research paradigm of music sociology. Although the current research still has limitations such as insufficient examination of music styles and lack of cross-cultural comparisons, it highlights the broad prospects for the deep integration of emotion computing, machine learning, and other artificial intelligence technologies with humanities and social science research. Future research can be deepened in the directions of incorporating more influencing factors, expanding cross-cultural perspectives, optimizing recognition models, and mining associations between emotions and behaviors, continuing to explore the rich emotional landscape and profound social connotations contained in pop music, and providing new insights for understanding the symbiotic relationship between music and society, technology and humanities.

## Data Availability

The raw data supporting the conclusions of this article will be made available by the authors, without undue reservation.

## Footnotes

1 30-s segments strike a balance: they are long enough to capture relatively stable emotional content which include the intro and early main theme, yet short enough to support fine-grained temporal analysis and efficient model inference.

2 In order to obtain reliable emotion labels at the song level, we conducted a manual annotation process based on the improved Thayer model. A total of 5 native Chinese annotators, all with at least 10 years of training or professional experience in musicology, psychology, or related fields and regular exposure to Chinese pop music, participated in the task. Before formal annotation, the annotators received a training session using 12 representative songs that covered all six emotion categories (Q1–Q6). We provided written guidelines that explained the semantic connotations of each category and offered typical examples. During the formal stage, each annotator independently assigned the dominant emotion category to the complete song, with the option of marking secondary emotions when necessary. To evaluate the reliability of the annotations, we calculated inter-annotator agreement (IAA) using Fleiss’ κ across all annotators and emotion categories. For songs with highly inconsistent labels, a consensus discussion was organized among the annotators, and a final label was determined by majority vote. This procedure ensured that the emotion labels used to train and evaluate the model are both conceptually consistent and statistically reliable.
